# Endosymbionts Alter Larva-to-Nymph Transstadial Transmission of *Babesia microti* in *Rhipicephalus haemaphysaloides* Ticks

**DOI:** 10.3389/fmicb.2018.01415

**Published:** 2018-06-27

**Authors:** Lan-Hua Li, Yi Zhang, Dan Zhu, Xiao-Nong Zhou

**Affiliations:** ^1^School of Public Health and Management, Weifang Medical University, Weifang, China; ^2^Key Laboratory of Parasite and Vector Biology, Ministry of Health, National Institute of Parasitic Diseases, Chinese Center for Disease Control and Prevention, Shanghai, China

**Keywords:** *Rhipicephalus haemaphysaloides*, endosymbiont, *Coxiella*, *Rickettsia*, *Babesia microti*, transmission capacity

## Abstract

Maternally inherited endosymbionts inhabit a variety of arthropods. Some of them can protect the arthropod host against a wide range of pathogens. However, very little is known about the association between endosymbionts and pathogen susceptibility in ticks. The present study investigated the effect of endosymbionts on larva-to-nymph transstadial transmission of *Babesia microti* by *Rhipicephalus haemaphysaloides* ticks. Engorged female ticks were injected with PBS, ciprofloxacin or kanamycin. The offspring larvae were used to infest *B. microti-*positive mice. Prevalence of *B. microti* among the nymphs in different treatment groups and its association with endosymbiont density in the larvae were analyzed. The results showed that the prevalence of *B. microti* in the kanamycin-treated group (63.9%, 95% confidence interval (CI): 52.8–75.0%) was higher than that in the PBS (23.6%, 95% CI: 13.8–33.4%) or ciprofloxacin-treated (25.0%, 95% CI: 15.0–35.0%) groups. This increased prevalence was associated with reduced density of *Coxiella*-like endosymbiont but was not related to the density of *Rickettsia*-like endosymbiont. No direct evidence has previously been reported about the impact of *Coxiella*-like endosymbiont on pathogen susceptibility in ticks. This study reveals that endosymbionts are potentially important defensive symbionts of *R. haemaphysaloides* which may influence the colonization or susceptibility of *B. microti* in the tick host.

## Introduction

Ticks are among the most important vectors of human and animal pathogens in the world. They can transmit various pathogens including viruses, bacteria, and parasites (Man et al., 2016; Ait et al., 2017; Zhuang et al., 2018). In addition to pathogens, a diverse group of symbiotic microorganisms are also present in ticks. To date, bacteria of the *Rickettsia*, *Coxiella*, *Francisella*, *Arsenophonus*, *Candidatus Midichloria mitochondrii*, and *Wolbachia* genera have been found to be symbiotic in ticks (Liu et al., 2013). Symbionts may play important roles in the host’s reproduction, development, nutritional adaptation, defense against environmental stress, and immunity (Dale and Moran, 2006). For example, *Coxiella*-like endosymbiont was revealed to be associated with reproductive fitness of *Amblyomma americanum* and *Haemaphysalis longicornis* (Zhong et al., 2007; Zhang et al., 2017), while *Ca. Midichloria mitochondrii* was found to play a role in the engorgement and molt of *Ixodes ricinus* ticks (Ninio et al., 2015). Bacterial symbionts can also influence pathogen acquisition, colonization or susceptibility in ticks (Narasimhan et al., 2014; Gall et al., 2016; Abraham et al., 2017). For example, antibiotic treatment could change the susceptibility of *I. scapularis* ticks to *Anaplasma phagocytophilum*, *A. marginale*, *Borrelia burgdorferi*, and *Francisella novicida* by disrupting the symbionts (Gall et al., 2016). However, the biology of symbionts and their effect on ticks is still largely unexplored (Ahantarig et al., 2013; Bonnet et al., 2017).

Human babesiosis is an important tick-borne zoonotic disease and is considered an emerging threat in China (Vannier and Krause, 2015). *Babesia microti* is one of the major pathogens of human babesiosis. *Rhipicephalus haemaphysaloides*, a widely distributed tick species in China (Chen et al., 2010; Yu et al., 2015), has been proven to be a vector of *B. microti* (Li et al., 2016). Our previous study revealed that *Rickettsia* and *Coxiella*-like endosymbionts (*Rickettsia*-LE and *Coxiella*-LE hereafter) inhabit *R. haemaphysaloides* ticks (Li et al., 2018). However, the potential effects of endosymbionts on pathogen susceptibility in this tick species have not been investigated before. Therefore, we explored the effects of endosymbionts on larva-to-nymph transstadial transmission of *B. microti* by antibiotic-treated *R. haemaphysaloides* ticks.

## Materials and Methods

### *Babesia microti*, Ticks and Experimental Animals

*Babesia microti* used in the present study was the Peabody mjr Strain (ATCC PRA-99), provided by Institute of Laboratory Animal Sciences, Chinese Academy of Sciences. An engorged female *R. haemaphysaloides* tick was initially removed from a dog in Tengchong County, Yunnan Province of China, and was then maintained in our laboratory at 25°C, with 85% relative humidity and a 14/10 h light/dark photoperiod regimen, as described previously (Li et al., 2016). This started the colony of *R. haemaphysaloides* in our laboratory. Specific-pathogen-free female BALB/c mice weighing 16–18 g were used to feed larvae and nymphs for maintenance of the colony. New Zealand white rabbits were used to feed adult ticks. The engorged female *R. haemaphysaloides* ticks used for antibiotic treatment in present study were the fourth generation of the colony. All animal experiments were performed according to the protocols approved by the Ethics Committee at the National Institute of Parasitic Diseases, Chinese Center for Disease Control and Prevention, based in Shanghai (Approval No. IPD-2016-12).

### Antibiotic Treatments of Engorged Female Ticks

Nine engorged female ticks were randomly assigned to three treatment groups and injected with ciprofloxacin (10 mg/ml), kanamycin (10 mg/ml) or PBS separately, as described previously (Li et al., 2018). In brief, engorged female ticks were injected at the hemocoel between the first and second legs using a Hamilton microinjection syringe (Hamilton Co., Bonaduz, Switzerland). The needle was left inside the tick body for at least 30 s and then withdrawn slowly. The doses used for injection were 1 μl of solution per 100 mg body weight of engorged ticks. This dosage was proven to be effective to reduce the density of *Rickettsia*-LE or *Coxiella*-LE by our previous study (Li et al., 2018). The injected ticks were then maintained in separate containers for oviposition and incubation.

### Quantitative Detection of *Rickettsia*-LE and *Coxiella*-LE in Unfed Larvae

From each of the nine engorged female ticks, DNA was extracted from a subset of 30 unfed larvae using the DNeasy Blood & Tissue Kit (Qiagen, Valencia, Germany) according to the manufacturer’s protocol. In each round of DNA extraction, one blank sample was included to assess contamination. DNA concentration was quantified by Thermo Nanodrop spectrophotometers (Thermo Fisher Scientific Inc., Wilmington, DE, United States), and all DNA samples were adjusted to equal molar concentrations before qPCR detection. Relative densities of *Rickettsia*-LE and *Coxiella*-LE in larvae were then analyzed by the SYBR Green qPCR method, as described previously (Li et al., 2018). The beta-actin biosynthetic (*actin*) gene fragment of *R. haemaphysaloides* ticks, citrate synthase (*gltA*) gene fragment of *Rickettsia*-LE and *16S rRNA* gene fragment of *Coxiella*-LE were qPCR-amplified separately (**Table 1**). Relative density of *Rickettsia*-LE or *Coxiella*-LE was defined as *Rickettsia gltA* or *Coxiella 16S rRNA* gene copies per tick actin gene copy (Li et al., 2018).

**Table 1:** Primers for PCR and qPCR amplification.

Organism	Target gene	Primer	Sequence (5′ to 3′)	Reference
*B. microti*	*18S rRNA* gene	Bab1A	GTCTTAGTATAAGCTTTTATACAGCG	Li et al., 2016
		Bab4A	GATAGGTCAGAAACTTGAATGATACATCG	
		Bab2A	CAGTTATAGTTTATTTGATGTTCGTTTTAC	
		Bab3A	CGGCAAAGCCATGCGATTCGCTAAT	
*R. haemaphysaloides*	*Actin* gene	Ractin-F	GTGCCCATCTACGAAGGTTAC	Li et al., 2018
		Ractin-R	CCATCTCCTGCTCGAAGTCC	
*Rickettsia*-LE	Citrate synthase gene (*gltA*)	gltA-F	TCCTACATGCCGACCATGAG	Li et al., 2018
		gltA-R	AAAGGGTTAGCTCCGGATGAG	
*Coxiella*-LE	*16S rRNA* gene	L-CoxF	TGAGTGTTGACGTTACCCACAG	Li et al., 2018
		L-CoxR	GCATTTCACCGCTACACCG	


The volume of each qPCR mixture was 15 μl, containing 7.5 μl qPCR Master Mix Plus (Takara Bio Inc., Shiga, Japan), 0.5 μl of each 5 mM primer, 1.5 μl DNA sample, and 5 μl of water. Four negative controls were used in each 96-well qPCR plate. All qPCR was performed under conditions of 95°C for 30 s, 40 cycles of 95°C for 5 s and 60°C for 30 s, with the fluorescence recorded at the annealing stage, using a CFX96 Real-Time PCR System (Bio-Rad Laboratories Inc., Hercules, CA, United States).

### Acquisition of *B. microti* by *R. haemaphysaloides* Larvae

The cryopreserved *B. microti* stabilates were balanced to room temperature. Donor BALB/c mice were injected intraperitoneally with 100 μl of the stabilates. At the peak time of infection, namely, on the 8th day after incubation, blood was collected from the infected donor mice. A thin blood smear was made with 1 μl of tail blood. The smears were examined under microscope after being fixed with methanol and stained with Giemsa solution. A total of 500 red blood cells were counted for each smear. Parasitemia density was defined as the number of *B. microti* protozoa per 100 red blood cells, namely, the percentage of infected red blood cells.

The *B. microti*-positive blood was diluted with PBS to a parasitemia density of 15%. Nine BALB/c mice were inoculated intraperitoneally with 100 μl of diluted blood. Four days after incubation, the parasitemia density of each mouse was examined. The mice were divided into three groups using stratified randomization according to parasitemia density. From each maternal tick, 200 larval ticks were used to infest a *B. microti* infected mouse. The mice were monitored every day, and the engorged larvae were collected after falling from the mice. On the 4th day of infestation, the parasitemia density of each mouse was examined again.

Engorged larvae from each treatment and maternal tick were placed in two different containers. Those gathered on the 4th day of infestation were weighed individually by a Sartorius CPA225D balance (Ratingen, France) and put together into one container, while those gathered on other days were put together into another container. Four weeks after the first molted tick was observed (Li et al., 2016), 24 nymphs were randomly selected for *B. microti* detection from the container with larvae engorged on the 4th day of infestation.

### Detection of *B. microti* Infection in Nymphs

DNA was extracted from each nymph, quantified and adjusted to equal concentrations as described above. Negative controls were used during DNA extraction. Nested PCR was then used to test for infection with a set of specific primers targeting the *18S rRNA* gene of *B. microti* (**Table 1**), under conditions of 3 min at 95°C, 35 cycles of 94°C for 40 s, 57 and 72°C for 30 s, and ending at 72°C for 5 min. The 20 μl PCR mixture contained 10 μl of 2× Taq PCR Master Mix with dyes (DBI Bioscience, Shanghai, China), 2 μl of each primer at 5 mM, 3 μl of DNA sample, and 5 μl of water. One positive and four negative controls were used in each 96-well PCR plate. DNA extracted from the blood of a *B. microti*-positive mouse was used as positive control, while DNA extracted from blank samples was used as negative control. The PCR was performed in a C1000 Touch^TM^ Thermal Cycler (Bio-Rad Laboratories Inc., Hercules, CA, United States). Samples with 154-bp PCR products were recognized as *B. microti*-positive. One positive PCR product was selected from each PCR plate for sequencing to confirm *B. microti* infection.

### Statistical Analysis

Statistical analysis was carried out with SPSS 19.0 software (IBM, Armonk, NY, United States). The relative densities of *Rickettsia*-LE and *Coxiella*-LE were log-transformed. One-way ANOVA was used to compare body weight of engorged larvae, parasitemia density of mice and log-transformed relative density of endosymbionts among groups, and the Student–Newman–Keuls (SNK) method was further used for pairwise comparison. The chi-square test was used to compare the nymphal prevalence of *B. microti* among different groups. Bonferroni method was used for pairwise comparison of prevalence. Spearman’s rank correlation was used to analyze the bivariate correlations among the parasitemia density of mice, weight of engorged larvae, density of endosymbionts, and prevalence of *B. microti* in nymphs. A multilevel fixed-effect logistic model was further used to analyze the separate effects of parasitemia density of mice, weight of engorged larvae, and density of endosymbionts on nymphal prevalence of *B. microti*. Significant differences were defined as *p* < 0.05 with a two-tailed test.

**FIGURE 1:**
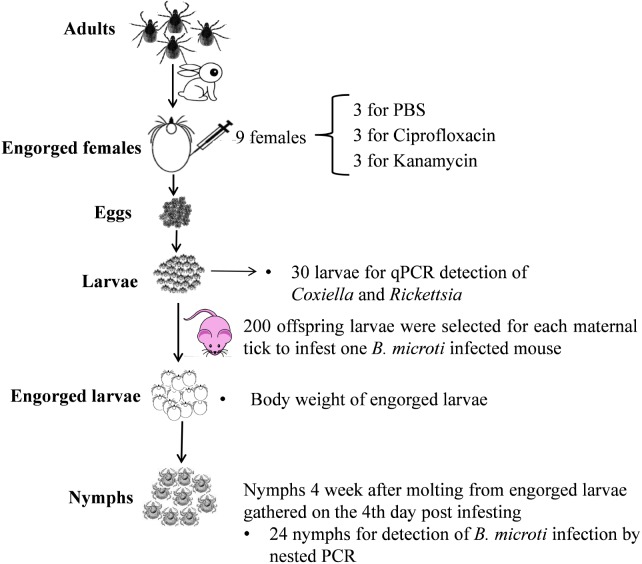
Larva-to-nymph transmission experiments of *B. microti* through endosymbiont-disrupted *R. haemaphysaloides* larvae.

The overall experimental procedure is shown in **Figure 1**.

## Results

### Effects of Antibiotic Treatment on the Density of *Coxiella*-LE and *Rickettsia*-LE in *R. haemaphysaloides* Larvae

**FIGURE 2:**
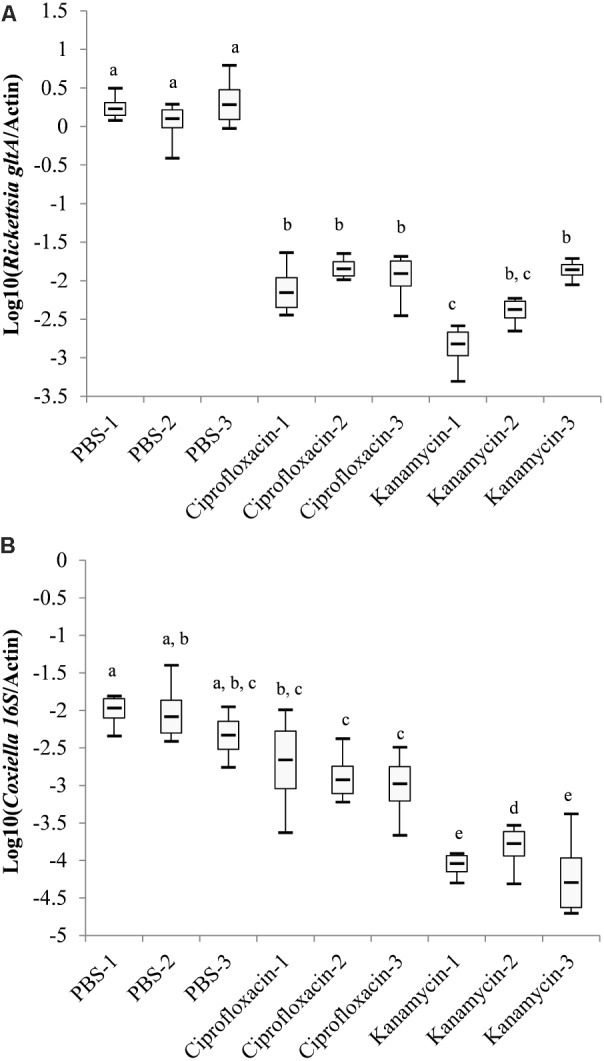
Log-transformed relative density of *Coxiella*-LE **(A)** and *Rickettsia*-LE **(B)** in *R. haemaphysaloides* larvae from the antibiotic- or PBS-treated groups (Groups with the same letters showed no statistical significance in pairwise comparisons).

Nine engorged female ticks were randomly divided into three equal groups and injected with PBS, ciprofloxacin or kanamycin. The relative density of *Rickettsia*-LE and *Coxiella*-LE in the offspring larvae were analyzed by qPCR (**Figure 2**). Pairwise comparison using the SNK method showed that the relative density of *Rickettsia*-LE in the larvae of both ciprofloxacin- and kanamycin-treated groups were statistically lower than that in the larvae of the PBS-treated group (**Figure 2A**). The relative density of *Coxiella*-LE was statistically lower in the kanamycin-treated group than in the ciprofloxacin- and PBS-treated groups; however, ciprofloxacin injection had little influence on the relative density of *Coxiella*-LE when compared to the PBS treatment (**Figure 2B**).

### Effects of Antibiotic Treatment on Larva-to-Nymph Transmission of *B. microti* by *R. haemaphysaloides*

The larvae of each maternal tick were used to infest *B. microti-*positive mice. For each maternal tick, 24 offspring nymphs were selected for *B. microti* detection from engorged larvae gathered on the 4th day of infestation. As shown in **Table 2**, the prevalence of *B. microti* in kanamycin-treated group (63.9%, 95% confidence interval (CI): 52.8–75.0%) was significantly higher than that in the PBS (23.6%, 95% CI: 13.8–33.4%), and ciprofloxacin-treated groups (25.0%, 95% CI: 15.0–35.0%). Meanwhile, prevalence among groups within the same treatment was not statistically different (Supplementary Table S1). *B. microti* infection was further confirmed by sequencing of PCR products.

**Table 2:** Prevalence of *B. microti* in *R. haemaphysaloides* nymphs from different treatment groups.

Treatment	No. of nymphs detected	No. positive	Prevalence, % (confidence interval)^1^	χ^2^	*P*
PBS	72	17	23.6 (13.8, 33.4)^a^	32.2	<0.001
Ciprofloxacin	72	18	25.0 (15.0, 35.0)^a^		
Kanamycin	72	46	63.9 (52.8, 75.0)^b^		


*^*1*^Groups with same letters means no statistical significance.*

### Impacts of the Parasitemia Density of Mice, Weight of Engorged Larvae, and Density of Endosymbionts on the Prevalence of *B. microti* in Nymphs

Both the body weight of engorged larvae and parasitemia densities of mice on the 1st and 4th day of infestation were similar among the different groups (*P* = 0.44, 0.96, and 0.92, respectively; **Tables 3**, **4**). As shown in **Table 5**, prevalence of *B. microti* in nymphs was negatively associated with relative density of *Coxiella*-LE in larvae (correlation coefficient = -0.87, *P* = 0.002) but not associated with relative density of *Rickettsia*-LE (correlation coefficient = -0.50, *P* = 0.175). According to the multilevel logistic regression model (**Table 6**), the altered density of *Coxiella*-LE affected the prevalence of *B. microti* (*P* = 0.002). This effect was present regardless of parasitemia density of mice, body weight of engorged larvae or relative density of *Rickettsia*-LE.

**Table 3:** Parasitemia density of *B. microti* in mice at different time points after infestation with PBS- or antibiotic-treated *R. haemaphysaloides* ticks.

Treatment	ID of mouse	Day 1 (%)^1^	Day 4 (%)^2^
PBS	1	23.4	66.8
PBS	2	17.3	58.5
PBS	3	12.9	59.9
Ciprofloxacin	4	20.8	69.3
Ciprofloxacin	5	16.5	62.8
Ciprofloxacin	6	13.2	57.7
Kanamycin	7	20.6	70.2
Kanamycin	8	19.7	65.8
Kanamycin	9	12.1	55.1


*^*1*^*P* = 0.96; ^*2*^*P* = 0.92.*

**Table 4:** Weights of engorged larvae gathered from different mice (micrograms).

Treatment	ID of mouse	No. of engorged larvae	Weight of engorged larvae ( 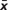 ± S)
PBS	1	24	0.302 ± 0.023
PBS	2	24	0.313 ± 0.021
PBS	3	24	0.305 ± 0.026
Ciprofloxacin	4	24	0.312 ± 0.025
Ciprofloxacin	5	24	0.306 ± 0.030
Ciprofloxacin	6	24	0.305 ± 0.022
Kanamycin	7	24	0.297 ± 0.024
Kanamycin	8	24	0.308 ± 0.019
Kanamycin	9	24	0.304 ± 0.022
*F* among treatments			0.8
*P* among treatments			0.44


**Table 5:** Bivariate correlation of parasitemia density of mice, body weight of engorged larvae, relative density of *Coxiella*- or *Rickettsia*-LE, and prevalence of *B. microti* in *R. haemaphysaloides* nymphs.

	Log-transformed relative density of *Coxiella*-LE	Prevalence of *B. microti* in nymphs
Log-transformed relative density of *Rickettsia*-LE	0.72^1^	-0.50
Log-transformed relative density of *Coxiella*-LE		-0.87^2^


*^*1*^*p* < 0.05, ^*2*^*p* < 0.01.*

**Table 6:** Potential factors associated with infection of *R. haemaphysaloides* nymphs as revealed by a two-level logistic regression model.

Variable	Coefficient	Standard error	*t*	*P*	95% CI
Intercept	-4.56	13.81	-0.33	0.74	-31.8, 22.7
Weight of engorged larvae	1.58	47.64	0.03	0.97	-92.4, 95.6
Parasitemia of mice on day 4 of infestation	0.00	0.04	0.01	0.99	-0.1, 0.1
Log-transformed relative density of *Rickettsia*-LE	0.26	0.24	1.05	0.30	-0.2, 0.7
Log-transformed relative density of *Coxiella*-LE	-1.29	0.41	-3.15	0.002	-2.1, -0.5


## Discussion

Maternally inherited symbionts can protect their arthropod hosts against a wide range of pathogens (Bonnet et al., 2017). For example, *Wolbachia* may disrupt the transmission of a variety of pathogens, including viruses, bacteria, protozoa, and nematodes, by the insect host. Recently, *Wolbachia* has been used to control mosquito-transmitted diseases (Hoffmann et al., 2011). In comparison, very little is known about the association between symbionts and pathogen susceptibility in ticks. Our previous study has revealed that both *Coxiella*-LE and *Rickettsia*-LE inhabit 100% of *R. haemaphysaloides* ticks (Li et al., 2018). Meanwhile, *Coxiella*-LE was found to be associated with reproduction in *R. haemaphysaloides* ticks, but *Rickettsia*-LE was not (Li et al., 2018). However, the effect of *Rickettsia* or *Coxiella* endosymbionts on pathogen susceptibility in *R. haemaphysaloides* ticks has not been well established. In the present study, we investigated the impact of *Rickettsia*-LE and *Coxiella*-LE on larva-to-nymph transstadial transmission of *B. microti* by *R. haemaphysaloides* ticks. The results showed that the density of *Coxiella*-LE was related to the susceptibility of ticks on *B. microti*, but *Rickettsia*-LE was not.

*Coxiella*-LE is found only in ticks and inhabits at least two-thirds of tick species (Duron et al., 2017). Despite its wide distribution in ticks, the effects of *Coxiella*-LE on pathogen transmission by ticks are still unknown. Klyachko et al. (2007) localized *Coxiella* endosymbiont within *A. americanum*. *Coxiella*-LE was observed in several tick tissues, including the midgut, and salivary gland. They speculated that *Coxiella*-LE might be involved in the transmission of other pathogens. However, no direct evidence has been reported about the impact of *Coxiella*-LE on pathogen transmission in ticks. Our previous study found that *Coxiella*-LE was the most abundant symbiont in *R. haemaphysaloides* ticks (Li et al., 2018). The present study further revealed that the reduced density of *Coxiella*-LE in larval ticks of *R. haemaphysaloides* is associated with larva-to-nymph transstadial transmission of *B. microti*. It has been argued that maternal antibiotic treatment might change the amount of blood ingested by the offspring larvae (Dai et al., 2010). Thus, we compared the parasitemia density of mice and body weight of engorged larvae among different groups, and we analyzed their influence on transstadial transmission of *B. microti* from larva to nymph. The results showed that the association between *Coxiella*-LE and the prevalence of *B. microti* existed regardless of the body weight of engorged larvae and parasitemia density of mice (**Tables 3**–**6**). The results suggest that *Coxiella*-LE may play an important role in limiting the infection of *B. microti* in *R. haemaphysaloides* ticks.

*Rickettsia*-LE is also very common in ticks and inhabits more than half of tick species (Duron et al., 2017). It is the second most abundant symbiont in *R. haemaphysaloides* ticks and occurs in 100% of ticks from this species (Li et al., 2018). Previous studies have suggested that *Rickettsia*-LE may influence pathogen susceptibility in ticks. Steiner et al. (2008) investigated coinfection of *A. phagocytophilum*, *B. microti*, *B. odocoilei*, *B. burgdorferi*, and *Rickettsia*-LE in field-collected questing *I. scapularis* ticks. They found that *Rickettsia*-LE and *B. burgdorferi* co-occurred less frequently than expected in male ticks. Macaluso et al. (2002) studied the ability of *Dermacentor variabilis* ticks to maintain multiple species of rickettsiae through transovarial transmission. They revealed that rickettsial infection inhibited transovarial transmission of another *Rickettsia* (Macaluso et al., 2002). Meanwhile, Gall et al. (2016) treated female *D. andersoni* ticks with an antibiotic and explored the pathogen–endosymbiont relationship in the offspring ticks. The results showed that an increase in the quantity of *Rickettsia*-LE was associated with reduced *A. marginale* levels in blood-fed ticks. The above studies suggested that *Rickettsia*-LE may be an important defensive symbiont protecting ticks against pathogens (Bonnet et al., 2017). However, the present study found that a reduced density of *Rickettsia*-LE was not associated with the susceptibility of *R. haemaphysaloides* ticks to *B. microti*. The results suggest that *Rickettsia*-LE and different pathogens may have varying interactions (Gall et al., 2016).

There are several limitations of the present study. First, in addition to endosymbionts, ticks can harbor various extracellular bacteria, especially in the guts (Abraham et al., 2017). It is possible that antibiotic treatment of the adult ticks could simultaneously change the abundance of other bacteria in the guts of the offspring larvae. Hence, the altered transmission capability might be derived from the impact of bacteria other than *Coxiella*-LE and *Rickettsia*-LE. However, since *Coxiella*-LE and *Rickettsia*-LE are transovarially transmitted, while extracellular bacteria are often obtained from the environment (Kwan et al., 2017), the impact of gut bacteria was not investigated in our study. More investigations are still needed to assess the effect of maternal antibiotic treatment on the composition of other bacteria, especially the gut microbiome, in offspring larvae. Second, only 24 offspring nymphs were tested for *B. microti* infection per female tick, and thus, the result of the present study provides a preliminary understanding of the role of endosymbionts on *B. microti* transstadial transmission in *R. haemaphysaloides*. Considering the similar prevalence among groups that received the same treatment and the significant differences in prevalence among different treatments (**Table 2** and Supplementary Table S1), we believe that the results of the present study are robust. Third, *Coxiella*-LE and *Rickettsia*-LE were not evaluated in each organ of the nymphs. Nevertheless, it is justified to consider that the density of these endosymbionts was altered in all relevant organs of the offspring ticks, because antibiotic treatment of maternal ticks reduces the number of endosymbionts in eggs (Li et al., 2018). Finally, *B. microti* infection was not tested in larvae engorged from positive mice or during the molting process. Instead, we assumed that the number of protozoa ingested by the larvae depended on the amount of blood being ingested by ticks and the parasitemia density of the blood, and our study revealed that the average body weight of engorged larvae and the parasitemia density of blood were similar among different treatment groups.

## Conclusion

Our study revealed that maternal kanamycin treatment could reduce the density of *Coxiella*-LE in the offspring larvae, thus leading to increased larva-to-nymph transstadial transmission of *B. microti* in *R. haemaphysaloides* ticks. However, a reduced density of *Rickettsia*-LE due to ciprofloxacin treatment was not associated with susceptibility of *R. haemaphysaloides* ticks to *B. microti*. The results indicate that *Coxiella*-LE is likely an important defensive endosymbiont, and it potentially can influence pathogen colonization or susceptibility in ticks. More investigations are needed to assess the influence of endosymbionts on nymph-to-adult transmission of *B. microti* in ticks, to observe the dynamics of *B. microti* protozoa during the molting process of engorged ticks and to further investigate the underlying defensive mechanisms of these endosymbionts.

## Author Contributions

L-HL designed and performed the experiments and drafted the manuscript. YZ, DZ, and X-NZ conceived the study and revised the manuscript. All authors read and approved the final manuscript. Written consent to publish was obtained.

## Conflict of Interest Statement

The authors declare that the research was conducted in the absence of any commercial or financial relationships that could be construed as a potential conflict of interest.
