# Supplementary material for: Endosymbionts Alter Larva-to-Nymph Transstadial Transmission of Babesia microti in Rhipicephalus haemaphysaloides Ticks
Source: Front Microbiol. 2018 Jun 27;9:1415. doi: 10.3389/fmicb.2018.01415 (PMC6036257; doi:10.3389/fmicb.2018.01415)
Supplement: Supplementary file 1 [file Table_1.DOCX]

Supplementary Material

Endosymbionts alter larva-to-nymph transstadial transmission of *Babesia microti* in *Rhipicephalus haemaphysaloides* ticks

Lan-Hua Li, Yi Zhang, Dan Zhu, Xiao-Nong Zhou^*^

*** Correspondence:** Xiao-Nong Zhou: [zhouxn1@chinacdc.cn](mailto:zhouxn1@chinacdc.cn)

# Supplementary Table

**Supplementary Table 1**. Prevalence of *B. microti* in *R. haemaphysaloides* nymphs within and among different antibiotic treatment groups

| Treatment | ID of mouse | No. of nymphs detected | No. of positive | Prevalence, %  (Confidence interval) | χ2 | P |
| --- | --- | --- | --- | --- | --- | --- |
| PBS | 1 | 24 | 5 | 20.8 (4.6, 37.1) | 0.6 ^1^ | 0.74 |
| PBS | 2 | 24 | 5 | 20.8 (4.6, 37.1) |  |  |
| PBS | 3 | 24 | 7 | 29.2 (11.0, 47.4) |  |  |
| Ciprofloxacin | 4 | 24 | 6 | 25.0 (7.7, 42.3) | 0 ^1^ | 1 |
| Ciprofloxacin | 5 | 24 | 6 | 25.0 (7.7, 42.3) |  |  |
| Ciprofloxacin | 6 | 24 | 6 | 25.0 (7.7, 42.3) |  |  |
| Kanamycin | 7 | 24 | 15 | 62.5 (43.1, 81.9) | 0.1 ^1^ | 0.94 |
| Kanamycin | 8 | 24 | 15 | 62.5 (43.1, 81.9) |  |  |
| Kanamycin | 9 | 24 | 16 | 66.7 (47.8, 85.5) |  |  |
| Total |  | 216 | 81 | 37.5 (31.0, 44.0) | 32.7 ^2^ | <0.001 |

^1^ Comparison within each treatment group; ^2^ Comparison among all the nine groups
